# Mu opioid receptor stimulation in the medial preoptic area or nucleus accumbens facilitates song and reward in flocking European starlings

**DOI:** 10.3389/fphys.2022.970920

**Published:** 2022-09-12

**Authors:** Brandon J. Polzin, Alyse N. Maksimoski, Sharon A. Stevenson, Changjiu Zhao, Lauren V. Riters

**Affiliations:** Department of Integrative Biology, University of Wisconsin-Madison, Madison, WI, United States

**Keywords:** positive reinforcement, negative reinforcement, social behavior, birdsong, affective state, communication, affiliation, songbirds

## Abstract

It has been proposed that social cohesion in gregarious animals is reinforced both by a positive affective state induced by social interactions and by the prevention of a negative state that would be caused by social separation. Opioids that bind to mu opioid receptors (MORs) act in numerous brain regions to induce positive and to reduce negative affective states. Here we explored a potential role for MORs in affective states that may impact flocking behavior in mixed-sex flocks of nonbreeding European starlings, *Sturnus vulgaris.* Singing behavior, which is considered central to flock cohesion, and other social behaviors were quantified after infusions of the MOR agonist D-Ala2, N-Me-Phe4, glycinol5-ENK (DAMGO) into either the medial preoptic area (POM) or the nucleus accumbens (NAC), regions previously implicated in affective state and flock cohesion. We focused on beak wiping, a potential sign of stress or redirected aggression in this species, to provide insight into a presumed negative state. We also used conditioned place preference (CPP) tests to provide insight into the extent to which infusions of DAMGO into POM or NAC that stimulated song might be rewarding. We found that MOR stimulation in either POM or NAC dose-dependently promoted singing behavior, reduced beak wiping, and induced a CPP. Subtle differences in responses to MOR stimulation between NAC and POM also suggest potential functional differences in the roles of these two regions. Finally, because the location of NAC has only recently been identified in songbirds, we additionally performed a tract tracing study that confirmed the presence of dopaminergic projections from the ventral tegmental area to NAC, suggesting homology with mammalian NAC. These findings support the possibility that MORs in POM and NAC play a dual role in reinforcing social cohesion in flocks by facilitating positive and reducing negative affective states.

## Introduction

Numerous studies on the neural control of social behavior focus on behaviors in reproductive contexts, including mating, bonding, and maternal behaviors. Although not as well studied, many animals also engage in important affiliative, non-sexual social behaviors outside a breeding context. This includes the remarkable flocking behaviors observed in many birds outside the breeding season (Emlen, 1952; Feare, 1984; Eens, 1997; Goodson and Kingsbury, 2011; Wilson et al., 2016; Riters et al., 2019). The formation of these flocks enhances safety and foraging efficiency (Powell, 1974; Lazarus, 1979; Sullivan, 1984; Thiollay and Jullien, 1998) and contributes to reproductive success through the development of social skills (Himmler et al., 2013; Vanderschuren and Trezza, 2014; Pellis and Pellis, 2017; Riters et al., 2017). It has been proposed that social cohesion in these flocks may be reinforced by both a positive (i.e., pleasurable) affective state induced by social interactions within the flock and by the removal of a negative (i.e., aversive) state that would be caused by social separation (Emlen, 1952; Riters et al., 2019). Here we define a rewarding a stimulus as one that elicits an approach response either because it induces pleasure or because it reduces an aversive state (White, 1989).

Opioids that bind to mu receptors both induce positive and reduce negative affective states (Matthes et al., 1996; Trescot et al., 2008; Jhou et al., 2012; Fields and Margolis, 2015), suggesting a potential role for opioids in reward associated with flocking behavior. This idea is supported by a growing number of studies that implicate mu opioid receptors (MORs) in behaviors considered critical for flock cohesion. For instance, peripheral injections of the opioid receptor antagonist naloxone in male zebra finches, *Taeniopygia guttata*, reduce the production of undirected song, a type of song proposed to promote flock cohesion (Khurshid et al., 2010). Likewise, in flocks of male and female European starlings, *Sturnus vulgaris,* peripheral administration of the MOR agonist fentanyl both facilitates singing behavior and reduces beak wiping (Stevenson et al., 2020), which in some species, including starlings, is considered a potential sign of stress or redirected aggression under stressful conditions (i.e., “displacement” behavior performed because an alternative response is prevented (Nephew and Romero, 2003; Bauer et al., 2011; de Bruijn and Romero, 2011; Lattin et al., 2017; Lattin et al., 2019)). Multiple studies using conditioned place preference (CPP) tests indicate that production of singing behavior in flocks of starlings and zebra finches is tightly coupled to an intrinsically-rewarded state (Riters and Stevenson, 2012; Riters et al., 2014; Hahn et al., 2017; Stevenson et al., 2020). Studies in starlings also demonstrate that this form of gregarious song is associated with opioid-mediated analgesia (i.e., the reduction of a negative state) (Kelm-Nelson et al., 2012). Together these studies suggest that opioids may act at MORs to promote flock cohesion by both inducing a positive affective state and reducing a negative affective state (Riters et al., 2019).

The medial preoptic area (commonly abbreviated POM in birds) is a critical site in which opioids induce both CPP and analgesia in rodents (Tseng et al., 1980; Agmo and Gomez, 1991; Tseng and Wang, 1992; Le Merrer et al., 2009). In starlings, correlational studies demonstrate positive associations between MOR mRNA in POM and song-associated reward, as measured using CPP tests (Riters et al., 2014). More recently causal studies demonstrate that experimental siRNA downregulation of MORs in POM suppresses gregarious singing behavior and disrupts song-associated CPP (Stevenson et al., 2020). These CPP test results suggest that the reward state that accompanies singing behavior in flocks may function to reinforce flocking behavior (Riters et al., 2019). Although shy of statistical significance, there was also a clear trend for the siRNA downregulation of MORs to increase beak wiping (Stevenson et al., 2020). These studies raise the possibility that MOR in POM may both enhance positive and reduce negative states to facilitate and reward song and flock cohesion.

The POM directly accesses the canonical mesolimbic reward pathway via direct projections to the ventral tegmental area (VTA), which then projects to the nucleus accumbens (NAC) (Balthazart et al., 1994; Riters and Alger, 2004; Husband and Shimizu, 2011). The NAC is well known for its role in reward, but this region is also involved in responses to negative stimuli, learning, and motor activity (Parkinson et al., 1999; Salgado and Kaplitt, 2015). A more contemporary view is that NAC plays a critical role in promoting responses to positive and the avoidance of negative stimuli (Floresco, 2015). In rats, stimulation of MORs in NAC can induce reward (as measured using self-administration and CPP tests) (Olds, 1982; van der Kooy et al., 1982) and reduce stress-related behaviors (Zarrindast et al., 2008). It is thus possible that the MORs in NAC also play a dual role in rewarding, and thus reinforcing, flocking behavior by inducing positive and reducing negative affective states. Studies on starlings have begun for the first time to explore a role for the NAC in the regulation of gregarious song. Positive correlations were found in male starlings between gregarious song and the numbers of cells immunolabeled for FOS and ZENK (aka egr-1) in NAC (Polzin et al., 2021). A second study suggested these relationships to be causal, with a few (3 of 6) birds beginning to sing after infusion of the highly selective MOR agonist DAMGO into the NAC at the highest dose used in the study (Maksimoski et al., 2021). At this dose no effects were observed on beak wiping; however, this has not been tested thoroughly.

Unlike the POM, only recently has the NAC in songbirds begun to be studied. For many years the location of NAC in birds was unclear (Riters et al., 2022). Studies in pigeons, chicks, and now in songbirds delineate rostral, shell, and core subdivisions for NAC (Mezey and Csillag, 2002; Carrillo and Doupe, 2004; Reiner et al., 2004; Balint and Csillag, 2007; Balint et al., 2011; Husband and Shimizu, 2011; Gutierrez-Ibanez et al., 2016; Polzin et al., 2021). In the present study, we focused on the rostral portion of NAC identified by Reiner (Reiner et al., 2004), which in mammals contains shell- and core-like anatomical connections (Zahm and Heimer, 1993). The pharmacology study reviewed above demonstrated that MOR stimulation in this rostral portion of NAC induced locomotion and feeding, which is similar to what is observed in mammals (Maksimoski et al., 2021). Yet studies have not confirmed that this location in songbirds receives dopaminergic input from the ventral tegmental area, which is a key characteristic of NAC in mammals.

The goal of the present study was to provide insight into the hypothesis that MORs in the POM and NAC may promote gregarious behaviors and social cohesion by promoting positive and reducing negative affective states. We were also interested in the possibility that the two regions may play distinct roles in social behavior and affective states. To do this, we compared the effects of infusion of the MOR agonist DAMGO into POM or NAC on song and other social behaviors. We focused on beak wiping to provide insight into a presumed negative state and then used CPP tests to provide insight into the extent to which infusions of DAMGO into POM and NAC that stimulated song are rewarding. Finally, we performed a tract-tracing study to confirm the presence of dopaminergic projections to NAC from the ventral tegmental area to provide further insight into homology.

## Methods

All experiments were performed under animal care and use protocols approved by the University of Wisconsin Animal Care and Use Committee and according to guidelines of the National Institutes of Health.

### Effects of MOR stimulation in the POM and NAC on gregarious song and establishment of CPP

Twenty-two adult European starlings, *Sturnus vulgaris*, were used as experimental animals (16 males, six females). Fewer females than males were tested because fewer females sang during prescreening, which was required for inclusion in the experiment (as detailed below). All birds were captured in fall and winter months from a local farm on the west side of Madison, Wisconsin and housed in same-sex cages on 18L:6D for more than 6 weeks so birds would become “photorefractory”. Photorefractoriness is a characteristic of early fall wherein starlings begin to sing in large, mixed-sex flocks (Dawson et al., 2001). For this study, starlings were housed in indoor aviaries (2.13 × 2.4 × 1.98 m) with flock mates, so each aviary had a total of 8 birds. Following testing and subsequent removal of a bird from an aviary, a replacement bird was added to maintain 8 birds in each flock. The aviaries contained natural and artificial perches and the birds had *ad libitum* access to food, drinking water, and bathing water. During the 18 h of light, talk radio was played to acclimate birds to voices and extraneous noise.

This study was conducted from May 2021 to November 2021. Three researchers observed birds in flocks for 20 min a day, for five consecutive days to identify singing birds to be used as focal animals. Each focal bird was observed by only one observer from the beginning to the end of its testing to control for inter-observer variability within each experimental bird. At the beginning of each observation, an audio recording of starling song was played to facilitate singing behavior (Marius Travell, YouTube). Focal birds were not selected until at least three birds in the aviary sang for more than 3 days in a row. Once focal birds were selected, a cannula guide was surgically implanted to target either the POM or NAC (see below). Two birds from each aviary were selected to be tested on alternate days, and observers were intentional about leaving at least one singing starling in each aviary unmanipulated to help facilitate song from other birds. Observers were blind to the cannula location of the focal birds during their observations.

### Cannula surgery

For all surgeries, birds were anesthetized with isoflurane and placed into a stereotaxic apparatus (Kopf, Tujunga, CA, United States) with the beak approximately 45° below the horizontal plane of the ear bars, and a 26-gauge stainless steel cannula guide (C315G-5UP/S*pp*; Plastics One) was implanted in either the POM or NAC. Specific details of surgical procedures can be found in (Kelm-Nelson et al., 2013). For the POM, the rostrocaudal coordinate was placed 1 mm posterior to the stereotaxis zero. The lateral target was placed 0.5 mm from skull zero, either to the right or left hemisphere (the side of the hemisphere was counterbalanced across birds). The vertical target was placed 6.5 mm below the skull zero. For the NAC, the cannula targeted the rostral pole of the NAC (Reiner et al., 2004; Maksimoski et al., 2021; Polzin et al., 2021). The cannula guide was angled at 4.5° in the stereotaxis, and the rostrocaudal coordinate was placed 1.5 mm anterior to the stereotaxis zero. The lateral target was placed 0.5 mm to the left or right hemisphere and was similarly counterbalanced. The vertical target was placed 6.4 mm below the skull zero. The cannula was secured to the skull using screws and dental cement (clear Ortho-Jet powder combined with acrylic liquid; Lang Dental Manufacturing Company, Inc.). After surgery, birds were moved to a cage outside of the aviary for 2 days to recover.

### Pharmacological manipulations

Starlings were moved back to their original aviaries following recovery. Experimenters observed the birds until both focal birds sang for at least 2 days in a row. Focal birds were then treated and tested for 4 days, with each treatment day separated by at least one “treatment washout” day. Focal birds in the same aviaries were tested on alternate days so that only one bird per aviary was tested on a single day. First, a habituation “injection” was performed for each bird by placing an empty cannula in the cannula holder for the same amount of time as for treatment injections (∼5 min). This was performed to habituate the bird to being captured, anesthetized, and put in a temporary cage to recover. After habituation, each experimental sequence consisted of four treatments: vehicle (sterile saline, 0.85%; 0.50 μL), two doses of the MOR agonist D-Ala2, N-Me-Phe4, glycinol5-ENK (DAMGO; Sigma-Aldrich, catalog #100929-53-1; low dose, 2.5 μg; high dose, 25 μg (all dissolved in 0.50 μL sterile saline), and treatment with the MOR antagonist Cys-Tyr-D-Trp-Arg-Thr-Pen-Thr-NH(2) (CTAP; 3 μg dissolved in 0.50 μL sterile saline) immediately followed by the low dose of DAMGO. The latter manipulation was used to test for MOR specificity by determining the degree to which CTAP prevented DAMGO effects on behavior. For this manipulation the low dose of DAMGO was selected *a priori* based on a recent study in starlings that showed that this dose in NAC facilitated song (Maksimoski et al., 2021). Here we also added a higher dose of DAMGO (25 μg) because in the same study doses lower than 2.5 did not stimulate singing behavior (Maksimoski et al., 2021). Each starling received each treatment once in a counterbalanced order. Treatments were color-coded so the observer was blind to treatment conditions with the exception of the CTAP treatment due to the nature of the paired injection.

On each test day, injections began between 09:00 and 15:30; however, each focal bird was injected and observed within approximately the same time period each day to control for potential circadian variation in singing within an individual bird. Experimental birds were caught rapidly with a net and then anesthetized using isoflurane and oxygen with the aid of a nose cone. The dummy cannula was carefully removed, and a 33-gauge cannula was connected to PE50 tubing (catalog #C232CT, Plastics One) with the treatment solution drawn into it. A vacuum syringe (Hamilton) connected to a Nanomite Syringe Pump (Harvard Apparatus) injected 0.50 μL of the treatment solution over a 2 min period. Following the injection, the cannula was left in place for 3 min to allow for diffusion and equalization of pressure from the cannula tip. Infusion volume was confirmed by examining the movement of an air bubble in the tubing. After cannula removal, the cannula dummy was replaced, and the bird was placed in a draped cage for 15 min of recovery before being placed back into its home aviary.

### Behavioral observations

A researcher observed each focal bird for 20 min. Starlings produce long, complex songs that commonly begin with introductory whistles, followed by variable phrase types, clicks and trills (Adret-Hausberger and Jenkins, 1988; Eens, 1997; Hausberger et al., 1997; Alger et al., 2016). In this study we counted continuously: introductory whistles, song fragments [1-2 s variable songs], full songs [song longer than 2 s, past studies show this song on average to be approx. 25 s (Riters et al., 2000; Alger et al., 2016)]). We additionally recorded bouts of beak wiping (separated by 1 s); displacements (a focal bird approaches another bird and that bird leaves within 1 s); and flights to new perches.

### Conditioned place preference (CPP)

Our goal was to run CPP tests on the same birds that we observed singing. Cannula remain affixed to the skull firmly for 3-4 weeks, before they lose adhesion, presumably because birds are flying, exploring, and interacting freely in aviaries with branches and flock mates. In an effort to test the same birds after singing and for CPP we pilot tested and then ran what we found to be the most rapid CPP approach supported by past research (Bardo et al., 1995) (discussed further in the discussion section). We were unable to test four of the NAC birds because cannulae did not remain securely affixed to the skull, so only 6 NAC birds were tested.

**FIGURE 1:**
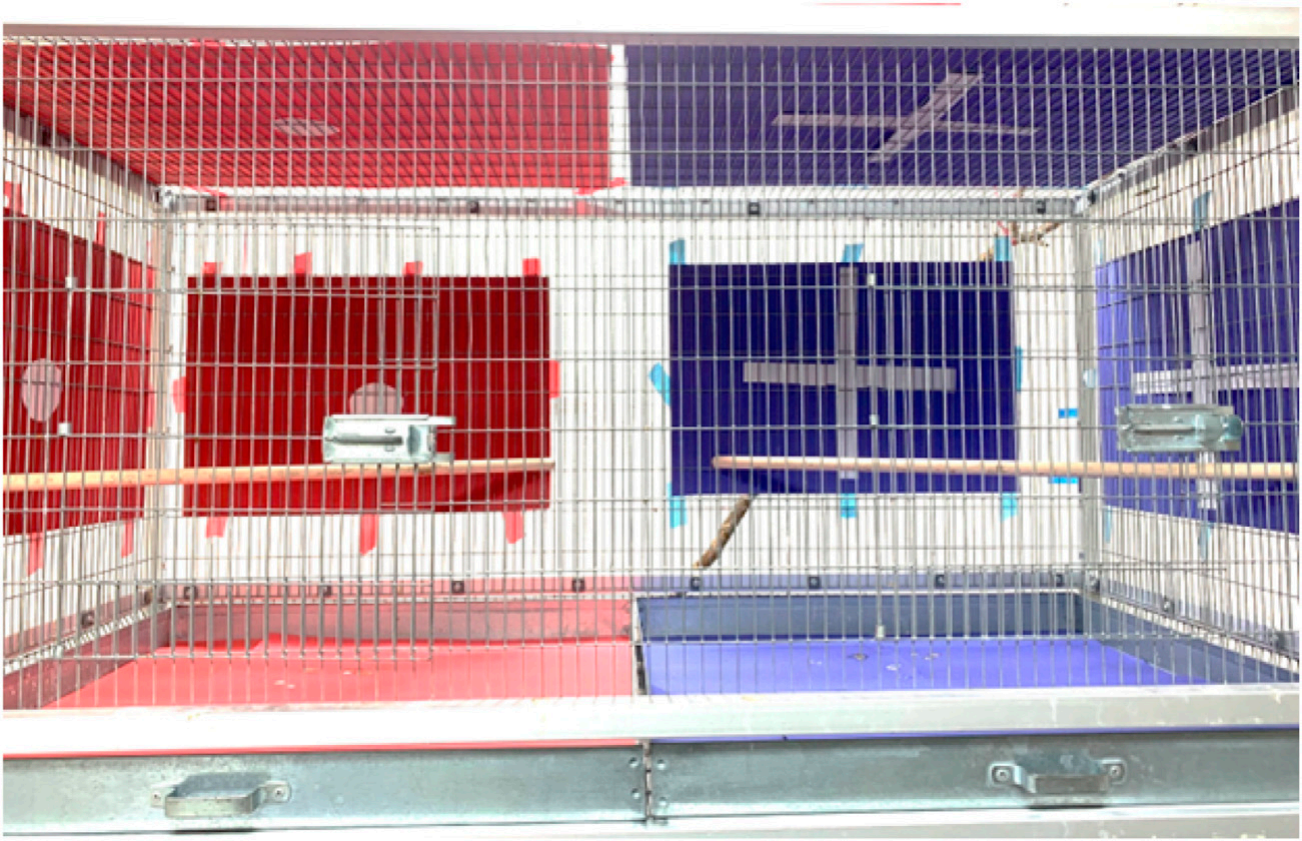
Photograph of the CPP apparatus to illustrate the two distinctly decorated conditioning compartments. On two separate days, birds were restricted to one side after saline treatment and to one side after DAMGO treatment (sides and treatments counterbalanced across birds). The next day a partition separating the two sides was removed (as in the photo) and the amount of time each bird spent on each side was recorded. See text for additional details.

CPP testing began at least 1 day after the final behavioral observation. The CPP apparatus consisted of a cage (118 cm × 59 cm × 59 cm) divided into two visually unique compartments with one compartment decorated with a red background with a white circle in the center and the other with a blue background with a white cross in the center (Figure 1). There were 3 phases each occurring on consecutive days: conditioning day 1, conditioning day 2, and test day. Observers were blind to the treatment conditions. On conditioning day 1, a focal bird was injected with either 0.5 μL of the low dose of 2.5 μg DAMGO or vehicle solution and allowed to recover using the above protocol (dose selected *a priori* based on (Maksimoski et al., 2021)). After recovery, the bird was placed singly into one side of the CPP apparatus and restricted to one of the distinct chambers for 45 min. Following conditioning, the bird was returned to its home aviary. The next day, conditioning day 2, the bird received whichever treatment it did not receive on conditioning day 1, recovered and was placed in the opposite, distinct compartment of the apparatus for 45 min and returned to its aviary afterward. The treatment order, side of the cage (left or right), and compartment (i.e., blue w/cross or red w/dot) paired with each treatment were all counterbalanced across birds.

On test day, the bird was caught and immediately placed in the center of the CPP apparatus with the divider of the two compartments removed, which allowed the starling to freely move between compartments. Lights were off in the room when the bird was placed in the center of the CPP apparatus, and lights were switched on to start the observation period. Observers then sat behind a one-way mirror and recorded how much time was spent on each side of the apparatus.

### Cannula tip verification

**FIGURE 2:**
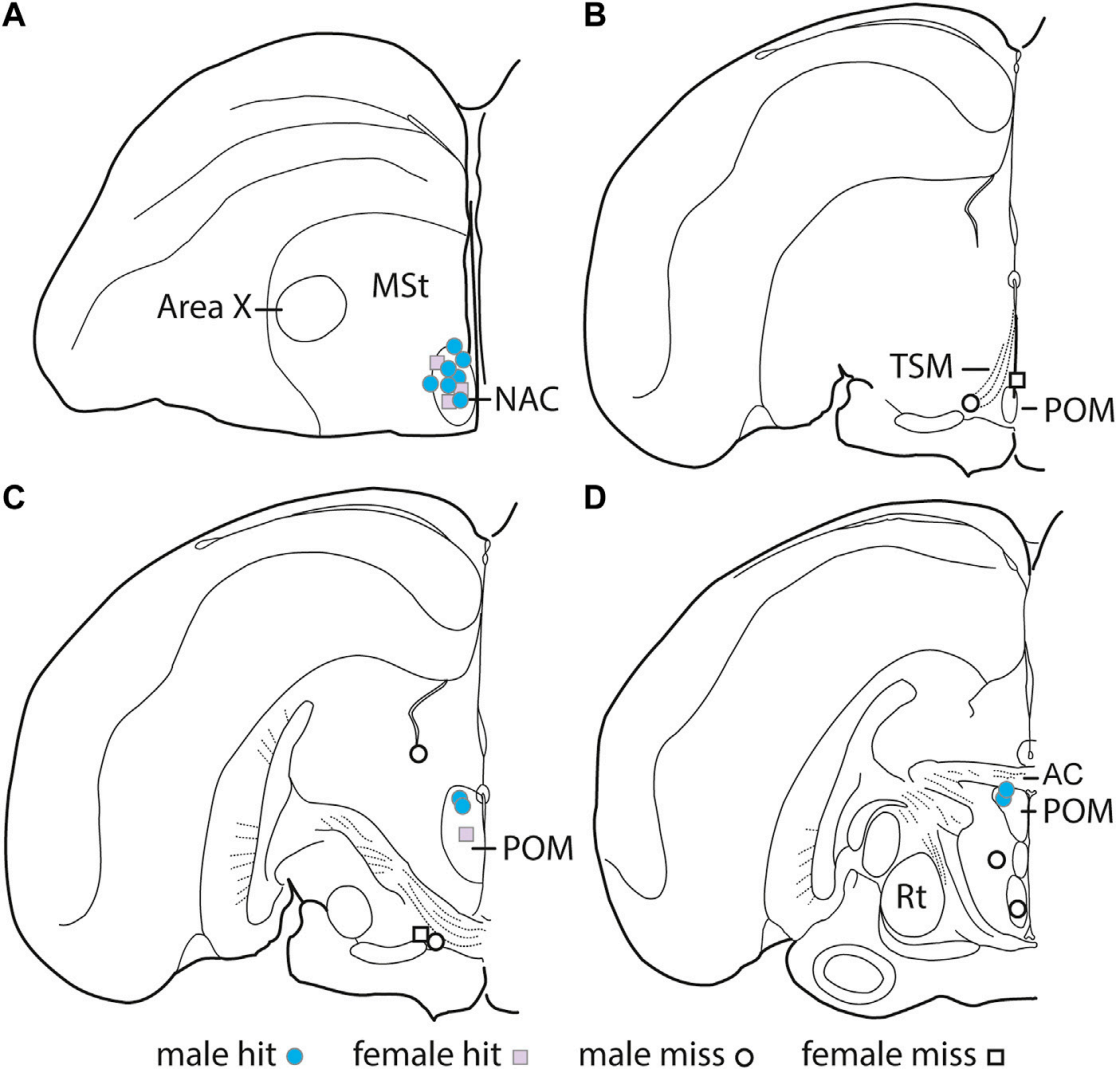
Location of DAMGO infusion sites. Illustration of one hemisphere of starling brain, with hits represented by filled-in shapes and misses represented by open shapes. Males are represented by circles, and females are represented by squares. AC, Anterior commissure; MSt, medial striatum; Rt, nucleus rotundus; TSM, tractus septomesencephalicus.

Immediately following the CPP test day observation, birds were anesthetized and injected with 1.0 ul of Chicago Blue 6B dye (Thermo Fisher Scientific) to identify the tip of the cannula. After infusion, the birds were sacrificed via rapid decapitation, brains were extracted and flash frozen with dry ice, and stored at −80°C. Brains were sectioned in 50 μm sections using a cryostat (catalog #CM 1850, Leica Biosystems). The sections were mounted on slides and analyzed using a microscope to determine whether the blue dye (i.e., the injection point) was accurately in the NAC or POM. There were 10 “hits” for the NAC (3 female, seven male) and 5 “hits” for the POM (1 female, four males) (Figure 2). Seven of the cannula tips were located outside of either NAC or POM (2 females, five males), which is common for stereotaxic surgery in wild birds. These “misses” were in multiple locations (Figure 2 and detailed in results).

### Identifying TH + projections to rostral NAC from the VTA

#### Retrograde tracing

We selected the rostral NAC identified by Reiner as the site of infusion for this study (Reiner et al., 2004). To explore further the degree to which this brain region is homologous to mammalian NAC, a tract-tracing study was performed in four birds (3 males, one female) that were not behaviorally tested to reveal the extent to which our NAC target received dopaminergic input (indicated by tyrosine hydroxylase [TH] labeling) from the ventral tegmental area (VTA), which characterizes NAC in mammals (Koob, 1992; Fields et al., 2007; Beier et al., 2015). Birds were anesthetized with isoflurane, and 0.5 μL of the retrograde tract tracer Fluoro-Gold (FG, Fluorochrome Inc., Denver, CO) was injected into NAC using identical methods as described above for cannula surgery. After infusion, starlings were returned to a cage for 10 days to allow for retrograde transport.

#### Double fluorescence immunohistochemistry for FG and TH with tyramide signal amplification (TSA)

Birds were anesthetized with isoflurane and transcardially perfused with 4% paraformaldehyde in 0.1 M phosphate buffer (PB; pH 7.4). Brains were post-fixed overnight in the same fixative and then cryoprotected with 0.1 M PB containing 30% sucrose at 4°C for 2 days. Brains were snap-frozen and 40-µm coronal sections were sliced on a cryostat and stored in cryoprotectant solution at −20°C until processing. Sections from just rostral to Area X through the VTA and periaqueductal gray were collected.

Brain sections were washed 5 × 5 min with 0.02 M PBS to remove cryoprotectant, and then incubated in 1.5% H_2_O_2_/50% methanol for 30 min to inhibit endogenous peroxidase activity and enhance the penetration of antibodies into tissue sections. They were rinsed 3 × 10 min in 0.3% Triton X-100/0.05% normal goat serum (NGS)/0.02 M PBS, incubated for 60 min in blocking solution (10% NGS/0.3% Triton X-100/0.02 M PBS), and then incubated overnight with a mixture of two primary antibodies: rabbit anti-FG (Fluorochrome, LLC, Denver; diluted 1:1000) and mouse anti-TH (MAB318, Millipore, Bilerica, MA, United States; diluted 1:1000) at 4°C in primary antibody incubation solution (PAIS, 0.3% Triton X-100/1% NGS/1% blocking reagent/0.02 M PBS). After primary antibody incubation, sections were washed and incubated for 1 h with HRP-conjugated goat anti-rabbit antiserum (Cell Signaling; diluted 1:100 in TBST), washed 3 × 10 min with wash buffer, then incubated for 10 min in Cy3-conjugated tyramide (TSA™ Plus Cyanine 3 kit, PerkinElmer, Waltham, MA; red for FG labeling) by diluting TSA stock solution 1:50 in 1x Amplification Diluent. After washing 3 × 10 min with wash buffer, sections were again incubated for 30 min with 3% H_2_O_2_ in TBS to quench peroxidase activity from the initial TSA reaction, incubated for 1 h with HRP-conjugated horse anti-mouse antiserum (Cell Signaling; diluted 1:100 in TBST). They were washed 3 × 10 min with wash buffer and then incubated for 30 min in Alexa Fluor 488-conjugated tyramide (Molecular Probes, Eugene, OR; green for TH labeling) by diluting TSA stock solution 1:100 in 1x Amplification reagent. Following washing 3 × 10 min with wash buffer, sections were mounted onto slides using DePeX mounting medium (Serva, Heidelberg, Germany), air-dried, and stored in the dark at 4°C. Cells double labeled for FG and TH in VTA, which demonstrates dopaminergic projections from VTA to TH, were detected on images taken using an inverted Zeiss LSM 710 Meta laser scanning confocal microscope (Zeiss; Oberkochen, Germany).

### Statistical analysis

Although both females and males were included in this study, during pre-tests fewer females sang in the aviaries, resulting in too few females in the study for statistical comparisons between sexes. However, we indicate the sexes in the figures. Sphericity was tested using Mauchly’s W. Assumptions of homogeneity of variance and normality were tested using Levene’s tests and Q-Q plots. When data violated assumptions, they were transformed using Log(x+0.05). In cases for which assumptions were not violated or for which transformation corrected violations parametric repeated measures ANOVAs were run with the behavioral variable entered as a dependent measure, treatment (saline, low dose DAMGO, high dose DAMGO, and CTAP + low dose DAMGO) entered as a repeated measures variable and region (POM or NAC) entered as a between-subjects variable. Significant ANOVAs were followed by pairwise post-hoc Tukey tests. When transformation did not correct violations, non-parametric Friedman ANOVAs were run. When significant, these were followed by pairwise Durbin-Conover post-hoc comparisons. Analyses were run using jamovi 2.3.5. Figures were made using the ggplot2 package in R v4.1.2 (Wickham, 2016; R Core Team, 2021).

## Results

Cannulae successfully targeted POM in 5 birds (4 males, 1 female) and NAC in 10 birds (7 males, 3 females). Cannulae missed the targets in 7 birds (5 males, 2 females) We briefly describe our results of cannula that missed the POM or NAC “misses” separately below.

### Singing behaviors

Two birds in the NAC group (1 male and 1 female) never sang again during experimental observations after surgery. These two birds were removed from analysis resulting in n = 8 birds in the NAC group for song analyses.

#### Introductory whistles

**FIGURE 3:**
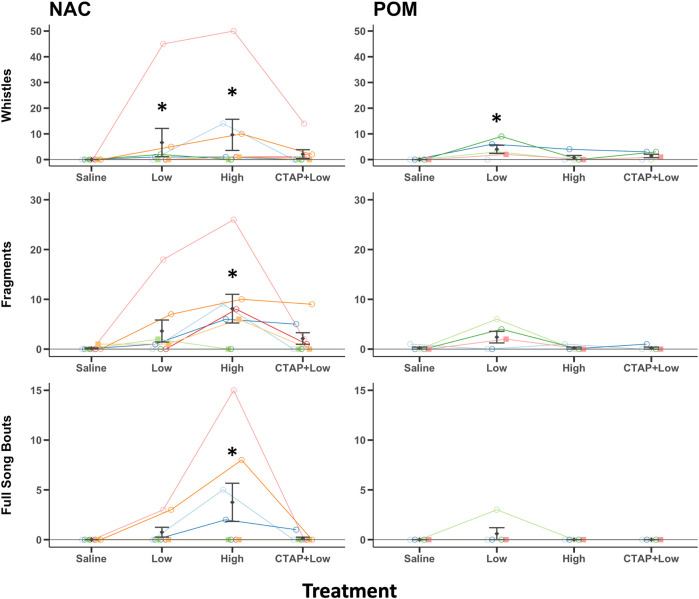
MOR stimulation in NAC or POM facilitates singing behaviors. Effects of intra NAC and intra POM treatments of Saline, low dose DAMGO (2.5 μg; Low), high dose DAMGO (25 μg; High) and the MOR antagonist CTAP followed by low dose DAMGO (CTAP + Low) on introductory whistles, song fragments, and full song bouts. Note: because whistle rates were low in some birds, they are just visible above the 0 line; however, for NAC 0/8 saline treated birds, 4/8 low dose DAMGO treated birds, 6/8 high dose DAMGO treated birds, and 3/8 CTAP + DAMGO treated birds whistled. For POM the results were 0/5, 4/5, 1/5, and 3/5, respectively. Mean ± sem is indicated for each group. Individual repeated data points are shown for males (circles) and females (squares). * indicates *p* < 0.05 compared to saline. See results for additional statistical details.

Introductory whistle components of song were observed in 0/8 NAC saline treated birds, 4/8 NAC low dose DAMGO treated birds, 6/8 NAC high dose DAMGO treated birds, and 3/8 CTAP + DAMGO treated birds. For POM the results were 0/5, 4/5, 1/5, and 3/5, respectively. Results of Friedman ANOVAs (performed because data violated assumptions as described in methods above) revealed significant effects of DAMGO treatment in NAC (χ^2_3_^ = 11.9, *p* = 0.008) and POM (χ^2_3_^ = 9.88, *p* = 0.020) (Figure 3). For NAC, post hoc Durbin-Conover tests indicate that compared to saline, birds whistled more after treatment with the low (*p* = 0.022) and high (*p* < 0.001) doses of DAMGO, but the low and high doses did not differ significantly (*p* = 0.077). Whistling was also significantly lower in the CTAP + DAMGO treatment compared to the high dose of DAMGO (*p* = 0.005) but was not significantly different from either saline (*p* = 0.228) or the low dose of DAMGO (*p* = 0.228) (Figure 3). For POM, post hoc Durbin-Conover tests indicate that compared to saline, birds whistled more after treatment with the low (*p* < 0.001) but not high (*p* < 0.381) doses of DAMGO and that the low and high doses differed significantly (*p* = 0.003). Whistling after the CTAP + DAMGO treatment was significantly lower relative to the low dose DAMGO treatment (*p* = 0.018) but did not differ from saline (*p* = 0.094) or the high dose of DAMGO (*p* = 0.381) (Figure 3).

#### Fragments

Fragments of song were observed in 1/8 NAC saline treated birds, 5/8 NAC low dose DAMGO treated birds, 6/8 NAC high dose DAMGO treated birds, and 4/8 CTAP + DAMGO treated birds. For POM the results were 1/5, 3/5, 1/5, and 1/5, respectively. Results of Friedman ANOVAs revealed significant effects of DAMGO treatment in NAC (χ^2_3_^ = 11.73, *p* = 0.008) but not for POM (χ^2_3_^ = 2.25, *p* = 0.522) (Figure 3). For NAC, post hoc Durbin-Conover tests indicate that compared to saline, birds produced fragments more after treatment with the high (*p* < 0.001) but not low (*p* = 0.094) doses of DAMGO, and the low and high doses differed significantly (*p* = 0.016). Production of fragments was also significantly lower in the CTAP + low dose DAMGO treatment compared to the high dose of DAMGO (*p* = 0.008) but was not significantly different from either saline (*p* = 0.159) or the low dose of DAMGO (*p* = 0.773) (Figure 3).

#### Full songs

Full songs were observed in 0/8 NAC saline treated birds, 2/8 NAC low dose DAMGO treated birds, 4/8 NAC high dose DAMGO treated birds, and 1/8 CTAP + DAMGO treated birds. For POM the results were 0/5, 1/5, 0/5, and 0/5, respectively. Results of Friedman ANOVAs revealed significant effects of DAMGO treatment in NAC (χ^2_3_^ = 9.55, *p* = 0.023) but for POM only one bird sang full songs (i.e., a male sang 3 times after the low dose of DAMGO) so no analysis was run on POM (Figure 3). For NAC post hoc Durbin-Conover tests indicate that compared to saline, birds sang full songs more after treatment with the high (*p* = 0.002) but not low (*p* = 0.288) doses of DAMGO, and the low and high doses differed significantly (*p* = 0.028). Production of full song was also significantly lower in the CTAP + low dose DAMGO treatment compared to the high dose of DAMGO (*p* = 0.008) but was not significantly different from either saline (*p* = 0.591) or the low dose of DAMGO (*p* = 0.591) (Figure 3).

#### Beak wiping

**FIGURE 4:**
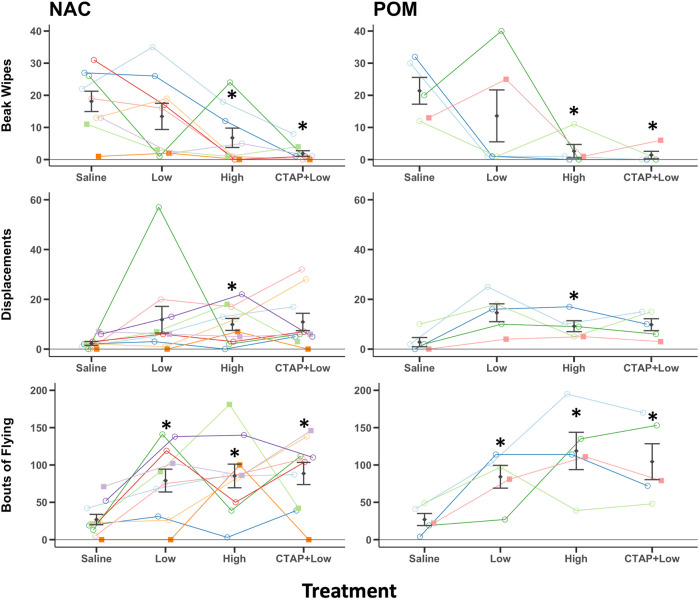
MOR stimulation in NAC or POM suppresses beak wiping but facilitates displacements and locomotion. Effects of intra NAC and intra POM treatments of Saline, low dose DAMGO (2.5 μg; Low), high dose DAMGO (25 μg; High) and the MOR antagonist CTAP followed by low dose DAMGO (CTAP + Low) on beak wiping, displacements, and flights. Mean ± sem is indicated for each group. Individual repeated data points are shown for males (circles) and for females (squares). Site of injection was not a significant factor, but we show results for NAC and POM separately here so points for each region can be seen clearly. * indicates *p* < 0.05 compared to saline for NAC and POM infusions combined. See results for additional statistical details.

The female that never sang again during experimental observations after surgery also failed to beak wipe and was removed from analysis, resulting in n = 9 birds in the NAC group for beak wipe analysis. For beak wiping, analysis of untransformed data revealed a significant main effect for treatment (F_3,36_ = 10.75, *p* < 0.001). No significant main effect was revealed for region (F_1,12_ = 0.01, *p* = 0.922) or the treatment x region interaction (F_3,36_ = 0.37, *p* = 0.776) (Figure 4). Post hoc Tukey tests indicate that compared to saline, birds beak wiped less after treatment with the high (*p* = 0.001) but not low (*p* = 0.539) doses of DAMGO. Beak wiping did not differ between low or high dose DAMGO treatments (*p* = 0.261). CTAP + low dose DAMGO treatment did significantly decrease beak wiping compared to saline (*p* < 0.001) and the low (*p* = 0.039) but not high (*p* = 0.467) doses of DAMGO (Figure 4).

### Displacements

For displacements, analysis of untransformed data revealed a significant main effect for treatment (F_3,39_ = 3.05, *p* = 0.040). No significant main effect was revealed for region (F_1,13_ = 0.019, *p* = 0.893) or the treatment x region interaction (F_3,39_ = 0.115, *p* = 0.951) (Figure 4). Post hoc Tukey tests indicate that compared to saline, birds displaced other birds more after treatment with the high (*p* = 0.025) but not low (*p* = 0.102) doses of DAMGO, but displacements did not differ between low or high dose DAMGO treatments (*p* = 0.858). CTAP + low dose DAMGO treatment did not significantly alter displacements compared to saline (*p* = 0.065), low (*p* = 0.926) or high (*p* = 0.991) doses of DAMGO (Figure 4).

### Flying

For flying, analysis of untransformed data revealed a significant main effect for treatment (F_3,39_ = 10.42, *p* < 0.001). No significant main effect was revealed for region (F_1,13_ = 0.825, *p* = 0.380) or the treatment x region interaction (F_3,39_ = 0.483, *p* = 0.696) (Figure 4). Post hoc Tukey tests indicate that compared to saline, birds flew to new perches more after treatment with the low (*p* = 0.002) and high (*p* = 0.001) doses of DAMGO, and flying did not differ between low or high dose DAMGO treatments (*p* = 0.677). CTAP + low dose DAMGO treatment increased flying compared to saline (*p* < 0.001) but did not alter flying relative to birds treated with the low (*p* = 0.777) or high (*p* = 0.987) doses of DAMGO (Figure 4).

### Conditioned place preference for DAMGO

**FIGURE 5:**
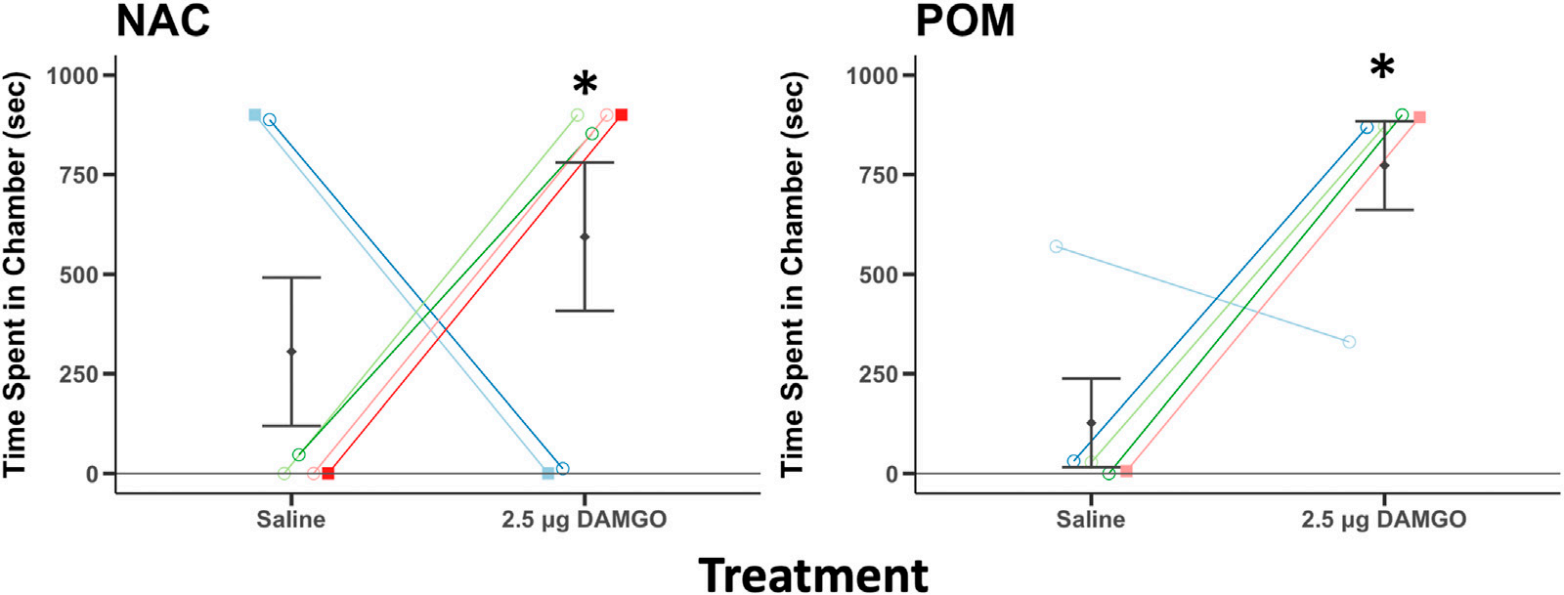
MOR stimulation in NAC and POM induces a conditioned place preference. Time spent in a chamber that had been paired previously with intra NAC or intra POM infusions of saline and 2.5 μg DAMGO. Mean ± sem is indicated for each group. Individual repeated data points are shown for males (circles) and for females (squares). * indicates that the difference score between the time spent by each individual on the side that had been paired with 2.5 DAMGO minus the side that had been paired with saline differed significantly from zero (*p* < 0.05). Note: for analysis we subtracted the time spent on the saline side from the time spent on the DAMGO paired side and compared this statistically to zero (which would reflect no preference). We present here the individual data points to provide readers with information on each individual. See results for additional statistical details.

In the two compartment CPP test used in this experiment the time on the previously saline paired side of the apparatus is reciprocally related to the time on the previously DAMGO paired side of the apparatus. Thus, the two variables were not sampled independently as required to run paired t-tests. To address this issue, we subtracted the time spent on the saline side from the time spent on the DAMGO paired side for each bird. If birds do not demonstrate a preference the difference would be zero while more positive scores reflect DAMGO-induced place preferences. We then ran single sample t-tests to compare the difference scores statistically to zero. A single sample *t*-test revealed that the difference scores for birds receiving DAMGO into NAC and into POM were significantly above zero for NAC (t_5_ = 0.3.19, *p* = 0.024) and for POM (t_4_ = 6.97, *p* = 0.002) (Figure 5), indicating DAMGO-induced place preferences.

### Results for infusions that missed NAC or POM

The “misses” were in multiple locations. One was in the third ventricle, one in the lateral ventricle, one in the tractus septomesencephalicus (TSM), two were ventral to intermediate portions of POM labeled as ventrolateral thalamus (VLT) in the Stokes et al. canary brain atlas (Stokes et al., 1974), one targeted the lateral hypothalamus (LH), and one targeted the ventromedial portion of the hypothalamus (VMH); Figure 2)). Statistical analyses were not run on the “misses” due to low sample sizes (n = 1 for most regions; Figure 2). We report behavioral results in Table 1 but do not discuss these results further.

**TABLE 1:** Effects on behavior of DAMGO infusions outside NAC or POM.

Song (whistle, fragments, full)			—		
Sex	Region	Saline	Low DAMGO	High DAMGO	CTAP + Low DAMGO
Male	TSM	0	3,3,0	2,16,1	0
Female	3rd vent	7,0,5	3,0,0	9,0,0	0,0,0
Male	lat. vent	0	0	0	0
Male	VLT	0	0	0	0
Female	VLT	0	0	0	0
Male	LH	0	0	1,0,0	3,0,0
Male	VMH	1,0,2	0	0	0
Beak wiping					
Male	TSM	6	3	0	0
Female	3rd vent	10	1	0	0
Male	lat. vent	4	8	0	0
Male	VLT	30	22	0	0
Female	VLT	51	0	0	0
Male	LH	3	17	1	0
Male	VMH	1	0	0	0
Displacement					
Male	TSM	0	9	11	21
Female	3rd vent	2	10	18	29
Male	lat. vent	4	0	6	7
Male	VLT	8	9	133	62
Female	VLT	1	0	0	0
Male	LH	2	4	10	3
Male	VMH	2	14	57	94
Flying					
Male	TSM	6	72	152	202
Female	3rd vent	26	105	174	152
Male	lat. vent	36	22	113	40
Male	VLT	43	19	229	96
Female	VLT	43	0	0	55
Male	LH	62	100	175	133
Male	VMH	37	100	120	162
**CPP**					
Sex	Region	Secs saline side	Secs DAMGO side	Difference score	—
Male	TSM	307	593	286	—
Female	3rd vent	900	0	−900	—
Male	lat. vent	470	430	−40	—
Male	VLT	326	574	248	—
Female	VLT	0	900	900	—
Male	LH	0	900	900	—
Male	VMH	752	148	−604	—

### Tract tracing and tyrosine hydroxylase labeling

**FIGURE 6:**
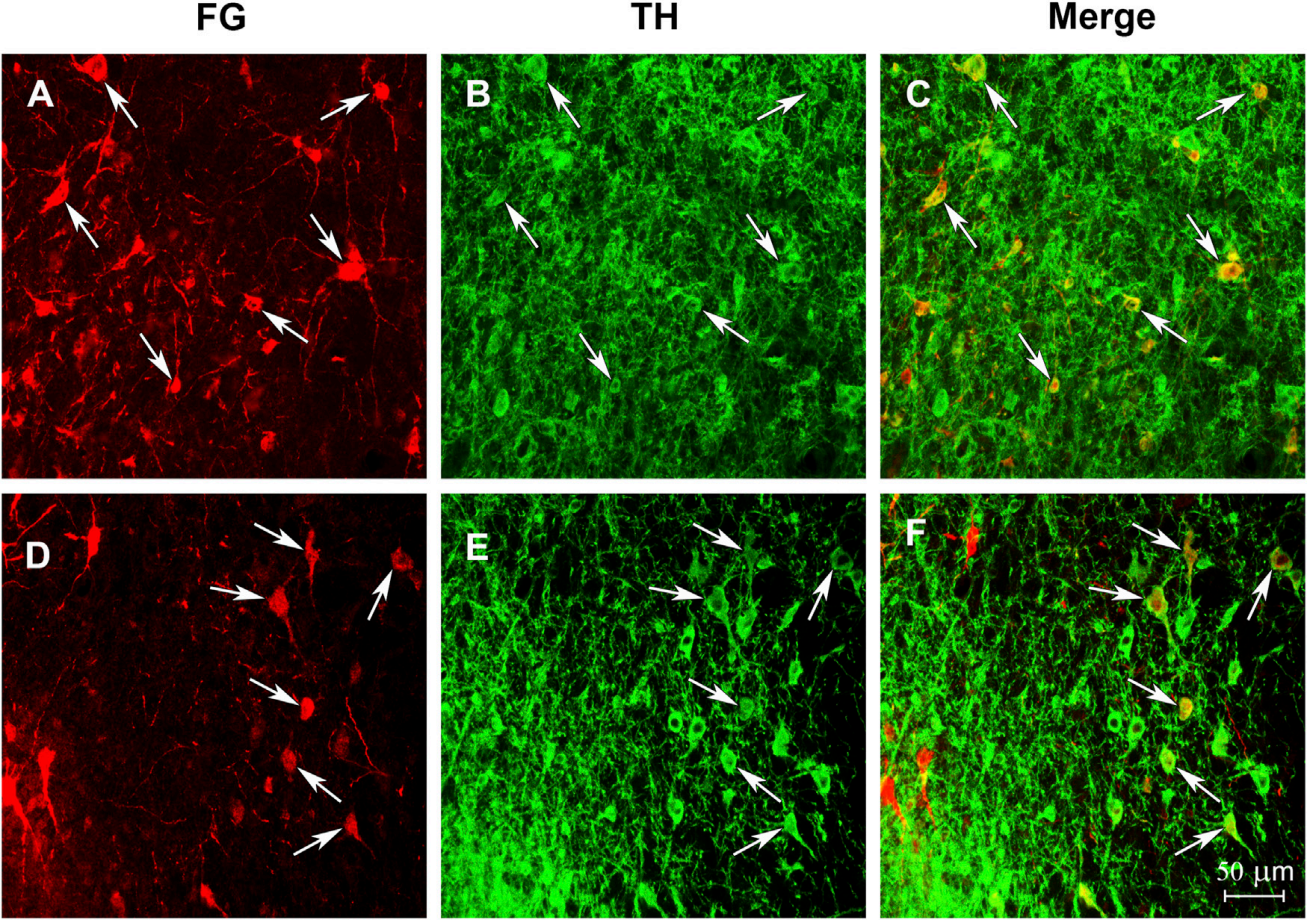
Photomicrographs illustrate that the starling NAC receives dopaminergic input from VTA. Confocal images showing immunolabeling in the VTA from two birds (one bird per row) that received injections of the retrograde tract tracer Fluro-Gold into the NAC. **(A and D)** illustrate neurons labeled with tracer. **(B and E)** illustrate neurons labeled for TH. **(C and F)** illustrate neurons double labeled for both the tracer and TH. Arrows highlight the same double labeled cells in each image. Left is lateral for each image.

After infusions of the retrograde tract tracer Fluro-Gold (FG) into NAC, many FG-positive cells were observed, with several cells double labeled for FG + TH being found within the ventral tegmental area in all four birds (Figure 6).

## Discussion

The results of this study demonstrate a causal role for MORs in both the NAC and POM in the facilitation of gregarious song, the reduction of a potentially negative state reflected in beak wiping, and reward demonstrated by the CPP tests. Collectively, these findings are consistent with the possibility that MORs in NAC and POM may play a dual role in flock cohesion by both reducing negative and inducing positive affective states. During pre-tests, few females sang frequently enough to be included in the study, similar to past studies on MOR in POM and MOR in NAC (Stevenson et al., 2020; Maksimoski et al., 2021), thus females could not be compared statistically to males. However, when results of all the studies to date that include males and females are considered together there do not appear to be any obvious sex differences in the effects of DAMGO in either region on behavior. This is somewhat expected given that the song produced by males and females in this context likely plays a shared function related to group cohesion.

### Mu opioid receptor stimulation in NAC or POM increases singing

Infusion of the selective MOR agonist DAMGO into NAC or POM stimulated singing behaviors differentially across doses, with subtle differences observed for distinct components of song. Specifically, the low dose of DAMGO in POM and both low and high doses of DAMGO in NAC stimulated low levels of introductory whistles, while song fragments and full songs were stimulated more robustly by the high dose of DAMGO in the NAC only. These effects were reversed by treatment with the MOR antagonist CTAP, indicating that effects on behavior were specific to MORs in both the NAC and POM.

Although birds produced few full songs after any of the treatments, song in this context is highly sensitive to disruption, and birds stop singing when caught and injected (Stevenson et al., 2020; Maksimoski et al., 2021); therefore, restoring full song production with injections of DAMGO into NAC is striking. When specific components of song were considered, DAMGO treatment in either NAC or POM was found to robustly increase the production of song fragments. This component of starling song consists of the production of short 1–2 s fractions of song referred to as warbling or variable song types (Adret-Hausberger and Jenkins, 1988; Eens, 1997). The function of fragments is not known; however, it is possible that the ongoing production of these short songs functions to maintain social cohesion or contact, similar to simple contact calls produced by numerous species (Marler, 2004).

Although DAMGO only increased the number of introductory whistles in some birds from zero to one or two, we find it striking that none of the birds whistled after saline treatment while for the NAC infused group 4 of 8 whistled after the low and 6 of 8 whistled after the high dose of DAMGO. For POM, 4 of 5 birds also whistled after a low dose of DAMGO. Introductory whistles are components of song that are proposed to convey information about species and individual identity (Adret-Hausberger, 1989). They are produced spontaneously or in response to another individual (Adret-Hausberger, 1982; Feare, 1984; Eens et al., 1989). Whistles increase seasonally in fall after the breeding season as starlings form and maintain overwintering flocks, and this component of song may promote flock cohesion by allowing birds to recognize other starlings and individuals within flocks (Adret-Hausberger, 1982; Adret-Hausberger, 1984). Collectively, these results establish a role for MOR in NAC in song production that was hinted at in a prior study (Maksimoski et al., 2021) and demonstrate effects to be dose dependent.

It was somewhat surprising to find that DAMGO in POM did not significantly facilitate the production of fragments or full songs given that MOR downregulation in POM suppresses singing behavior (Stevenson et al., 2020). This finding certainly does not preclude a role for MOR in POM in these components of song but suggests that NAC may be a more sensitive target for opioid stimulation of song in this context.

The POM is found to access directly regions underlying vocal production in passerines (i.e., “the song control system”) through a projection to the dorsomedial part of the nucleus intercollicularis and to indirectly access this system through projections to the ventral tegmental area, the periaqueductal gray, and the locus coeruleus (Riters and Alger, 2004). The pathway by which the NAC accesses the song system has not been studied; however, in mammals NAC is commonly considered a central limbic-motor interface (e.g. (Morrison et al., 2017)). NAC is reciprocally connected to both the POM and the VTA (Groenewegen and Russchen, 1984; Heimer et al., 1991; Zahm and Brog, 1992; Smeets and Medina, 1995; Salgado and Kaplitt, 2015; Ma et al., 2020), offering indirect routes by which NAC can impact singing behavior. These and other pathways can be identified in future studies.

### Mu opioid receptor stimulation in NAC or POM reduces beak wiping

The increases in singing behavior induced by DAMGO in NAC and POM were accompanied by stepwise, dose-dependent decreases in beak wiping, which is a proposed reflection of a negative state of stress or redirected aggression in starlings (Bauer et al., 2011; de Bruijn and Romero, 2011), and no differences in effects were observed between the two regions. One interpretation is that the presence of flock mates naturally leads to the release of opioids that bind to MOR in NAC and POM to reduce a negative state that facilitates singing and flock cohesion. However, unexpectedly, the DAMGO-induced suppression of beak wiping was not reversed, but in fact appeared to be enhanced, by pre-treatment with the MOR antagonist CTAP, suggesting effects may not be MOR specific. There is some precedent in the literature for this with administration of low doses of opioid receptor antagonists found to enhance effects of the MOR agonist morphine on analgesia (Crain and Shen, 2000). These effects appear to be caused by low doses of the antagonists preferentially inhibiting excitatory opioid receptors, which then enhances effects of activation of inhibitory opioid receptors by MOR agonists (Crain and Shen, 2001). If this is the case, higher doses of CTAP may be needed to observe the expected reversal of DAMGO effects. The degree to which effects are specific to MOR needs to be determined in future studies.

### Mu opioid receptor stimulation at a dose that facilitates song also induces a CPP

In addition to influencing song and beak wiping, strong conditioned place preferences were induced by infusions of the low dose of DAMGO (dose selected prior to the experiment based on a past study suggesting that this was a dose in NAC capable of triggering song (Maksimoski et al., 2021)). CPP tests are classic tests used to evaluate the rewarding properties of drugs of abuse (Bardo and Bevins, 2000), and the present findings are consistent with CPP studies in rodents that demonstrate that MOR stimulation in NAC and enkephalin infusion in POM induce strong place preferences (van der Kooy et al., 1982; Agmo and Gomez, 1991). Positive results in rodents have been taken as evidence that MOR stimulation in POM or NAC is rewarding (van der Kooy et al., 1982; Bardo et al., 1995; Olmstead and Franklin, 1997), although there are studies that do not show this effect for NAC, which may reflect discrepancies in injection sites (Bals-Kubik et al., 1993; Olmstead and Franklin, 1997). This reward may be induced by a pleasurable outcome or by the reduction of an aversive state, and CPP tests do not distinguish between the two (Ikemoto, 2007; McKendrick and Graziane, 2020). With respect to drugs that stimulate MORs, experiences in humans suggest that likely both states are induced.

Past studies in songbirds show clear linear, positive correlations between gregarious song and an individual’s intrinsic reward state, as measured using CPP tests (Riters and Stevenson, 2012; Riters et al., 2014; Hahn et al., 2017; Stevenson et al., 2020), and it has been proposed that either a reward state induces singing behavior or that the act of singing itself induces a reward state. Although the present findings do not rule out the latter interpretation, the finding that MORs in the POM or NAC induce CPPs in starlings supports the interpretation that the induction of a reward state by MOR activation facilitates gregarious singing behavior. Together the DAMGO-induced increase in singing, CPP, and reduction in beak wiping reported here lend support to the proposal that social cohesion in these flocks may be rewarded both the induction of a positive state resulting from behaviors that occur within the flock and by the reduction of a negative state.

The CPP design we selected was based in part on a meta-analysis of CPP tests for opiates (Bardo et al., 1995), which suggested that two rather than 3 compartments for the apparatus is most effective, that a 45-min pairing is better than 30 min, and the omission of a preconditioning phase results in larger effects. The rationale for this relatively short test is that we have only 3-4 weeks after cannula surgery to complete the studies. Birds take time post-surgery to resume singing, therefore we had a very limited time in which to perform the CPP test. Our pilot tests and the results reported here indicate that DAMGO infusion can induce a CPP following this design; however, there is a large literature on CPP testing, best practices, and caveats, some of which recommend inclusion of a neutral chamber and pretesting for initial side preferences (Bardo and Bevins, 2000; Prus et al., 2009; McKendrick and Graziane, 2020). It is thus possible that results do not reflect rewarding effects of DAMGO; however, given that the present findings are consistent with opioid CPP as well as self-administration studies in rodents (van der Kooy et al., 1982; Agmo and Gomez, 1991; Prus et al., 2009) we consider reward to be a parsimonious interpretation.

### Mu opioid receptor stimulation in NAC or POM increases displacements

For both POM and NAC, high doses of DAMGO significantly increased displacements, and no differences were observed between the two regions. These effects were prevented by pre-treatment with CTAP, suggesting results are MOR selective. The results for NAC are consistent with a past paper in which the same low dose of DAMGO used here increased displacements in starlings (Maksimoski et al., 2021). Here we also report for the first time that MOR stimulation in POM also increases displacements. It is important to note that displacements in starling flocks are mildly agonistic and relatively non-threatening. Starlings do not maintain strong dominance hierarchies and are observed sharing food outside the breeding context. They tend to maintain approximately one body width of distance between themselves presumably to avoid being pecked by other birds. Therefore, the function of displacements has been proposed to relate to the optimization of social spacing (King, 1973; Maksimoski et al., 2021). The present findings suggest that activation of MORs in POM and NAC may play a role in optimizing social spacing to maintain flock cohesion.

### Mu opioid receptor stimulation in NAC or POM stimulates motor behaviors

Stimulation of MORs in NAC or POM increased motor behavior as reflected in numbers of flights to new perches, with no differences observed between the two regions. Similar to beak wiping, CTAP did not reverse motor behavior. The results for NAC are consistent with past studies in birds and mammals (Cunningham and Kelley, 1992; Maksimoski et al., 2021). Although we found no reports on the impact of MOR in POM on motor activity, prior studies have revealed a role for the POM in voluntary wheel running in mice (King, 1979; Grigsby et al., 2020; Ladyman et al., 2021).

### Behavioral and anatomical data suggest findings in birds may generalize to other vertebrates

Unlike the POM, only recently has the NAC in songbirds begun to be studied. The results of the tract-tracing study showed that after infusion of the retrograde tract tracer FG into NAC, FG + TH positive cells were identified in VTA. This confirms that our NAC target site receives dopaminergic input from the VTA, an essential characteristic of the NAC in mammals. The behavioral results also confirm that manipulations of MOR in songbirds induce similar effects to those observed in mammals, including a reduction in proposed stress-related behavior and increased motor behaviors, reviewed above. Moreover, stimulation of MOR in the starling NAC induced a CPP which is consistent with findings from several rodent studies (Olds, 1982; van der Kooy et al., 1982). Together the present findings add to prior studies that support homology between the avian and mammalian NAC (Panzica et al., 1996; Roselli et al., 1998; Balthazart and Ball, 2007; Voigt et al., 2007). Along with past studies supporting homology between the avian and mammalian POM (e.g. (Balthazart and Ball, 2007)), this suggests that findings in birds related to social reward and cohesion will generalize to mammals. We thus propose that the findings reported here for NAC and POM are revealing new information about critical mechanisms and circuitry by which opioids reinforce essential, positive, stress-reducing non-sexual social behaviors.

## Data Availability

The raw data supporting the conclusions of this article will be made available by the authors, without undue reservation.
